# Supplementary material for: Structural remodelling of the carbon–phosphorus lyase machinery by a dual ABC ATPase
Source: Nat Commun. 2023 Feb 22;14:1001. doi: 10.1038/s41467-023-36604-y (PMC9947105; doi:10.1038/s41467-023-36604-y)

Structural remodelling of the carbon-phosphorus lyase machinery by a dual ABC ATPase

Søren K. Amstrup[1, 2], Sui Ching Ong[1], Nicholas Sofos[1, 2], Jesper L. Karlsen[1], Ragnhild B. Skjerning[1], Thomas Boesen[3], Jan J. Enghild[1], Bjarne Hove-Jensen[1], and Ditlev E. Brodersen[1, *]

SUPPLEMENTARY INFORMATION

Supplementary Fig. 1. The C-P lyase pathway. Pathway for the breakdown of phosphonate compounds by the bacterial C-P lyase pathway. In all intermediates, the phosphorus atom originating from the phosphonate is shown in red and R- indicates a carbon moiety. In step 1, PhnI in the presence of PhnG, PhnH and PhnL catalyses the phosphonylase reaction whereby the adenine base of ATP is exchanged by the phosphonate compound, forming 5-triphosphoribosyl-α-1-phosphonate. Next, PhnM catalyses the removal of pyrophosphate producing 5-phosphoribosyl-α-1-phosphonate and PP_i (step 2). 5-phosphoribosyl-α-1-phosphonate is then the substrate for the S-adenosylmethionine (SAM) dependent C-P lyase reaction catalysed by PhnJ (step 3), which liberates the carbon moiety (R-H) and produces 5-phosphoribosyl-1-2-cyclic phosphate. In step 4, PhnP a phosphoribosyl cyclic phosphodiesterase, hydrolyses the C2 ester resulting in production of ribose 1,5-bisphosphate. In step 5, the ribosyl bisphosphate phosphokinase PhnN phosphorylates ribose 1,5-bisphosphate to PRPP consuming 1 ATP. The last two steps responsible for the formation of P_i are not catalysed by enzymes encoded in the phn operon. However, a phosphoribosyltransferase can convert a nucleobase (e.g., adenine) and PRPP to a nucleoside 5'-monophosphate (such as AMP) and PP_i (6). Finally, PP_i can then be hydrolysed by a diphosphatase to P_i.

Supplementary Fig. 2. Purification of Phn(GHIJ)$_2$K. a, SDS-PAGE gel showing various purification stages of the Phn(GHIJ)$_2$K complex, including following Source Q and the two peaks that arise during Mono Q separation (peak 1 and peak 2), as well as after the final gel filtration step. The gel is representative of three repeated experiments. **b,** 3D density maps of PhnK from 3D classification with signal subtraction of the Phn(GHIJ)$_2$K data. In red, the map of a 3D class of PhnK with improved density and in semi-transparent blue a similar map from 3D classification with an extra density at the nucleotide binding site. **c,** Superposition of Phn(GHIJ)$_2$ (blue) and Phn(GHIJ)$_2$K (green) structures shown as ribbons. The overall rmsd (all atoms) between the structures is 0.497 Å.

Supplementary Fig. 3. Cryo-EM analysis of Phn(GHIJ)$_2$K. Diagram showing the overall strategy used to sort picked particles for the Phn(GHIJ)$_2$K structure. Initial steps of 2D and 3D classification in RELION yielding 1,329,400 particles were followed by heterogeneous refinement in cryoSPARC, resulting in 901,800 particles used to generate a 2.2 Å map in RELION. The mask used for 3D classification with signal subtraction and without image alignment is shown as a semi-transparent surface over all maps. The Phn(GHIJ)$_2$ core complex is shown in blue and PhnK in red. Particle classes from 3D classification with signal subtraction and no image alignment used in 3D variability analysis are marked in rounded boxes. The final map generated after 3D variability analysis and homogeneous refinement is shown at the bottom of the figure with accompanying Guinier plot, particle directional distribution plot, FSC curve, and local resolution map.

Supplementary Fig. 4. Cryo-EM analysis of Phn(GHIJKL)$_2$ bound to AMPPNP. Diagram showing the overall strategy used to sort picked particles for the Phn(GHIJKL)$_2$ PhnK E171Q AMPPNP structure. The Phn(GHIJ)$_2$ core complex is shown in blue, PhnK in red, and PhnL in yellow. Initial 3D volumes without interpretable density (classes 3 and 4) are shown in separate colours. Particle classes used in the following steps are marked in rounded boxes. The final structure (2.08 Å) was determined by imposing C2 symmetry as shown at the bottom of the Fig. with accompanying Guinier plot, particle directional distribution plot, FSC curve, and local resolution map.

Supplementary Fig. 5. Comparison of the ATP binding site in PhnK between different structures. a, Full map density (top) and details of the PhnK ATP binding site (bottom) with accompanying ligand density of the Phn(GHIJKL)$_2$ PhnK E171Q AMPPNP structure. The Mg^{2+} ion shown in light blue, the catalytic water molecule and other water molecules coordinating the Mg^{2+} ion are shown as red spheres. The catalytic Glu/Gln171 (E171Q) and accompanying Gln90 residue

are shown as sticks. Electron potential density for nucleotide ligands, the Mg^{2+} ion, waters, and visible residues are shown. **b,** Like a, but for the $Phn(GHIJKL)_2$ WT ADP + Pi structure. **c,** Like a, but for the $Phn(GHIJK)_2$ WT ATP structure. **d,** Structural alignment of the dimeric ABC modules in the *Staphylococcus aureus* Sav1866 ABC transporter bound to AMPPNP (PDB ID: 2ONJ, green) and PhnK E171Q (red), both bound to AMPPNP. The nucleotide and residues involved in binding, are shown as coloured sticks. The transmembrane part of Sav1866 is in grey with the coupling helix indicated (cyan). **e,** Superposition of the $Phn(GHIJ)_2K$ (green) and $Phn(GHIJKL)_2$ (red) structures shown as ribbons. The overall rmsd (all atoms) between the structures is 0.307 Å.

Supplementary Fig. 6. Identification of PhnL and PhnK-PhnL sequence alignment. a, Top hits from MASCOT following total protein analysis of a purified sample prior ion-exchange chromatography. All the top hits are C-P lyase core complex components (PhnGHIJ), PhnK, or PhnL. **b,** Electrostatic surface potentials of the PhnK (top side) and PhnL (underside) dimers as observed in the structure of $Phn(GHIJKL)_2$ PhnK E171Q AMPPNP, shown as a semi-transparent surface on top of cartoon representations. Electrostatic potential scale is from -5.0 to +5.0. **c,** Structural sequence alignment of PhnK, PhnL and *S. aureus* Sav1866 (PDB ID: 2ONJ) with conserved residues marked in green boxes. The secondary structural elements of PhnK and PhnL are shown above the sequences and labelled. The regions containing the extended β hairpin of PhnL and the C-terminal domain are shown in yellow and red boxes, respectively.

Supplementary Fig. 7. Purification of the $Phn(GHIJKL)_2$ complex and identification of PhnL. a, Purification of $Phn(GHIJKL)_2$ from pRBS01 expressed in *E. coli* Lemo21 cells. Four discrete peaks identified after Mono Q ion exchange chromatography (Peaks 1-4) were separated and investigated individually by gel filtration chromatography, as indicated. **b**, All peaks contained PhnG, PhnH, PhnI, PhnJ and PhnK, but only Peak 1 contained PhnL as indicated by SDS-PAGE and nucleotide (as

suggested by the higher OD260/OD280 ratio in a). Peak 1 also contained uncleaved PhnK (PhnK-Strep), despite having been incubated with TEV protease. The gel is representative of two experiments for each of wildtype and the E171Q mutant. **c,** ATPase activity of $Phn(GHIJKL)_2$ wt and $Phn(GHIJ)_2K$ measured by separation of nucleotide species by ion-exchange chromatography after overnight incubation with ATP.

Supplementary Fig. 8. Particle sorting during cryo-EM analysis of $Phn(GHIJKL)_2$ under ATP turnover conditions. Diagram showing the overall strategy used to sort picked particles for the dataset of $Phn(GHIJKL)_2$ under ATP turnover conditions. The $Phn(GHIJ)_2$ core complex is shown in blue, PhnK in red, and PhnL in yellow. Initial 3D volumes without interpretable density (classes 1 and 4) are shown in separate colours. Particle classes used in the following steps are marked in black, rounded boxes. The working sets of particles used for the reconstruction of the individual functional states, $Phn(GHIJKL)_2$ WT ADP+P_i, $Phn(GHIJK)_2$ open, and $Phn(GHIJK)_2$ closed, are shown in orange, red or blue boxes, respectively. The resolution and final set of particles (after box expansion and curation) were $Phn(GHIJKL)_2$ WT ADP+Pi (1.9 Å, 222,056 particles), $Phn(GHIJK)_2$ ATP closed (2 Å, 81.605 particles), and $Phn(GHIJK)_2$ open (2.6 Å, 31,280 particles).

Supplementary Fig. 9. Final maps from cryo-EM analysis of $Phn(GHIJKL)_2$ under ATP turnover conditions. a, Final electron potential maps for the $Phn(GHIJKL)_2$ WT ADP+Pi (1.9 Å) structure with accompanying Guinier plots, particle directional distribution plots, FSC curves, and local resolution maps. **b,** Like a, but for the $Phn(GHIJK)_2$ closed (2.0 Å) structure. **c,** Like a, but for the $Phn(GHIJK)_2$ open (2.6 Å) structure. The $Phn(GHIJKL)_2$ WT ADP+Pi and $Phn(GHIJK)_2$ ATP closed maps were calculated with imposed C2 symmetry.

Supplementary Fig. 10. Cryo-EM density features and 2D classes. a, Details and density at the ATP binding site of PhnL in the $Phn(GHIJKL)_2$ WT structure with density for ATP, Mg^{2+}, and Phe11,

Gln97, and Glu175 of the A loop shown. **b,** Local resolution maps of the PhnK dimer in the $Phn(GHIJKL)_2$ WT ATP+Pi structure. The resolution interval covers 0.8 Å. **c,** Like b, but for the $Phn(GHIJK)_2$ WT ATP structure. **d,** 2D-classes of the particles used to generate the $Phn(GHIJK)_2$ open, $Phn(GHIJK)_2$ closed ATP, and $Phn(GHIJKL)_2$ WT maps. **e,** Close-up of selected 2D classes showing a side view that reveals the presence or absence of PhnL in the respective particles. Density elements of the C-P lyase $Phn(GHIJ)_2$ core complex, PhnK and PhnL are marked with arrows.

Supplementary Fig. 11. Ligand binding at the PhnI-PhnJ interface Zn^{2+} site. a, Details and coordination of the octahedral (six-coordinated) Zn^{2+} at the PhnI-PhnJ binding interface as found in the closed conformation of the $Phn(GHIJKL)_2$ structure. Water molecules are shown as red spheres and coordinating interactions with dashed lines. Density for the Zn^{2+} and all coordinating ligands are shown. **b,** Interactions and EM density of the 5-phospho-α-D-ribose-1,2-cyclic-phosphate (PRcP) ligand at the PhnI-PhnJ interface binding site shown in two perpendicular orientations. Protein residues and backbones involved in the interaction with the ligand are shown as sticks and specific interactions are shown with dashed lines. Relevant residues as well as the 5' and 1'-2' cyclic phosphate groups are labelled. **c,** Binding pocket at the PhnI-PhnJ interface. The substrate binding pocket is shown with 5-phospho-α-D-ribose-1-phosphonate (PRPn) docked and carrying a phenyl R-group showing that the substrate cavity provides room for larger moieties. **d,** Coordination of the tetrahedral (4-coordinated) Zn^{2+} at the PhnI-PhnJ binding interface in the open conformation of the $Phn(GHIJK)_2$ structure. Density for the Zn^{2+} and all coordinating ligands are shown. **e,** Overview of the distance between Gly32 in PhnJ, which has been proposed to carry a radical as part of the reaction, the surface of the open cavity (10 Å) and the Zn^{2+} ion (31 Å).[1]

Supplementary Table 1. Cryo-EM data collection, refinement, and validation statistics

	$Phn(GHIJ)_2K$ (WT)	$Phn(GHIJKL)_2$ PhnK-E171Q AMPPNP	$Phn(GHIJKL)_2$ (WT) ADP+P_i
PDB accession ID	7Z19	7Z16	7Z15
EMDB accession ID	EMDB-14445	EMDB-14442	EMSB-14441
**Data collection and processing**			
Magnification	135,000	130,000	130,000
Voltage (kV)	300	300	300
Electron exposure (e–/Å^2)	~52	~62	~60
Defocus range (μm)	0.7-2.0	0.5-1.4	0.5-1.4
Pixel size (Å)	0.83	0.647	0.647
Symmetry imposed	C1	C2	C2
Initial particle images (no.)	901,800	1,023,261	874,692
Final particle images (no.)	50,323	59,737	222,056
Map resolution (Å)	2.57	2.08	1.93
FSC threshold	0.143	0.143	0.143
Map resolution range (Å)			
**Refinement**			
Initial model used (PDB ID)	4XB6	4XB6	4XB6
Model resolution (Å)	2.8	2.2	2.0
FSC threshold	0.50	0.50	0.50
Model resolution range (Å)			
Map sharpening *B* factor (Å^2)	-54.2	-28.8	-33.2
Model composition			
Non-hydrogen atoms	34,111	44,722	46,182
Protein residues	2174	2882	2888
Ligands	0	2	6
B factors (Å^2)			
Protein	84.45	35.05	21.80
Ligand	-	35.48	27.75
R.m.s. deviations			
Bond lengths (Å)	0.005	0.003	0.011
Bond angles (°)	0.627	0.633	0.954
Validation			
MolProbity score	1.48	1.28	1.16
Clashscore	6.60	4.78	3.76
Poor rotamers (%)	0.28	1.04	0.83
Ramachandran plot			
Favored (%)	97.4	97.9	98.4
Allowed (%)	2.6	2.1	1.6
Disallowed (%)	0.0	0.0	0.0

	**Phn(GHIJK)$_2$ (WT) ATP closed**	**Phn(GHIJK)$_2$ (WT) ATP open**
PDB accession ID	7Z18	7Z17
EMDB accession ID	EMDB-14444	EMSB-14443
**Data collection and processing**		
Magnification	130,000	130,000
Voltage (kV)	300	300
Electron exposure (e–/Å^2)	~60	~60
Defocus range (μm)	0.5-1.4	0.5-1.4
Pixel size (Å)	0.647	0.647
Symmetry imposed	C2	C1
Initial particle images (no.)	874,692	874,692
Final particle images (no.)	81,605	31,280
Map resolution (Å)	1.98	2.57
FSC threshold	0.143	0.143
Map resolution range (Å)		
**Refinement**		
Initial model used (PDB ID)	4XB6	4XB6
Model resolution (Å)	2.0	2.7
FSC threshold	0.50	0.50
Model resolution range (Å)		
Map sharpening *B* factor (Å^2)	-31.1	-28.7
Model composition		
Non-hydrogen atoms	37,705	37,614
Protein residues	2432	2432
Ligands	4	1
B factors (Å^2)		
Protein	147.68	39.82
Ligand	31.49	23.47
R.m.s. deviations		
Bond lengths (Å)	0.005	0.002
Bond angles (°)	0.870	0.467
Validation		
MolProbity score	1.18	1.52
Clashscore	3.31	6.55
Poor rotamers (%)	1.19	0.84
Ramachandran plot		
Favored (%)	98.1	97.1
Allowed (%)	1.9	2.9
Disallowed (%)	0.0	0.0

Supplementary Table 2. Plasmids used in this work

Name	Insert	Derived from vector	Marker
pHO575	*phnGHIJK* PhnK C-terminal 6xHis		Ampicillin
pRBS01	*phnGHIJKLMNOP* PhnK C-terminal TEV-2xStrep	Insert: pBW120<br>Vector: pET28a	Kanamycin
pRBS01-PhnK E171Q	*phnGHIJKLMNOP* PhnK C-terminal TEV-2xStrep	pRBS01	Kanamycin
pRBS01-PhnL E175Q	*phnGHIJKLMNOP* PhnK C-terminal TEV-2xStrep	pRBS01	Kanamycin
pRBS01-PhnK E171Q-PhnL E175Q	*phnGHIJKLMNOP* PhnK C-terminal TEV-2xStrep	pRBS01-PhnK E171Q	Kanamycin
pSKA03	*phn* operon: PHO-box + *phnC-P*	Insert: pBW120<br>Vector: pUC18	Ampicillin

Supplementary Table 3. Primers used for plasmid construction. For cloning primers (RBS01-RBS08), template-specific sequences are in italics, while for mutagenesis primers (SKA01-SKA16, SCO01-SCO02), mutations are indicated by underlined sequences.

Name	Sequence (5'-3')
RBS01	GTCGCGGTATCTGCGTG*CATGGTATATCTCCTTCTTAAAGTTAAACAAAATTATTTCTAGAGGGG*
RBS02	TGGAGATTGGGGTGGCGTG*ACAAAGCCCGAAAGGAAGC*
RBS03	TGTCATCGGTTTTGCAGAA*TGAAAACCTGTACTTCCAGGGTCAATTCG*
RBS04	ACGCCGCATCCGGCACCGGC*TTATTTTTCGAACTGCGGGTGGCTCCACGA*
RBS05	CCCTGGAAGTACAGGTTTTC*ATTCTGCAAAACCGATGACACC*
RBS06	CTTTAAGAAGGAGATATACC*ATGCACGCAGATACCGC*
RBS07	CAGCTTCCTTTCGGGCTTTG*TCACGCCACCCCAATCTCCA*
RBS08	ACCCGCAGTTCGAAAAATA*AGCCGGTGCCGGA*
SKA01	ATGGATCAGCCGACCGGCGGGCTGGATGTG
SKA02	TCGGCTGATCCATAAACACCAGCTTCGGATGCGTC
SKA03	CCTAACCCTCTCCCCAGAGG*TCTAGAGTCGACCTGCAGGCATG*
SKA04	GAAGAGTTAACGGGAATTCG*GGATCCCCGGGTACCGAGC*
SKA05	AGCTCGGTACCCGGGGATCCCGAATTCCCGTTAACTCTTCATCTGTTAGTCAC
SKA06	GCCTGCAGGTCGACTCTAGACCTCTGGGGAGAGGGTTAGGGTG
SKA07	CTGGAAGCGTACGGCGTGATGCAGGTGAAACTGTATG
SKA08	CCGTACGCTTCCAGCGCGTGCATGGTGCGGG
SKA09	GAAACTGGCGGAAGATATCGCCCGCTTCGGTCATATCG
SKA10	GATATCTTCCGCCAGTTTCACCTGCATCACGCCGTAC
SKA11	GCGCGCGCCTGCTGGCGACCGAATGGGGCGTGGTGCATCAG
SKA12	GGTCGCCAGCAGGCGCGCGCGGTCGGCCTCGCTCATTGCATAC
SKA13	ATGGATCAGCCGACCGGCGGGCTGGATGTG
SKA14	TCGGCTGATCCATAAACACCAGCTTCGGATGCGTC
SKA15	CTTGACCAGCCTACCGCCTCGCTGGACGCCAAAAAC
SKA16	GTAGGCTGGTCAAGCAGCAGAATGGGGTAGTCG
SCO01	CTTGACCAGCCTACCGCCTCGCTGGACGCCAAAAAC
SCO02	GTAGGCTGGTCAAGCAGCAGAATGGGGTAGTCG

References

1. Kamat SS, Williams HJ, Dangott LJ, Chakrabarti M, Raushel FM. The catalytic mechanism for aerobic formation of methane by bacteria. *Nature* **497**, 132-136 (2013).

Supplementary Figure 1

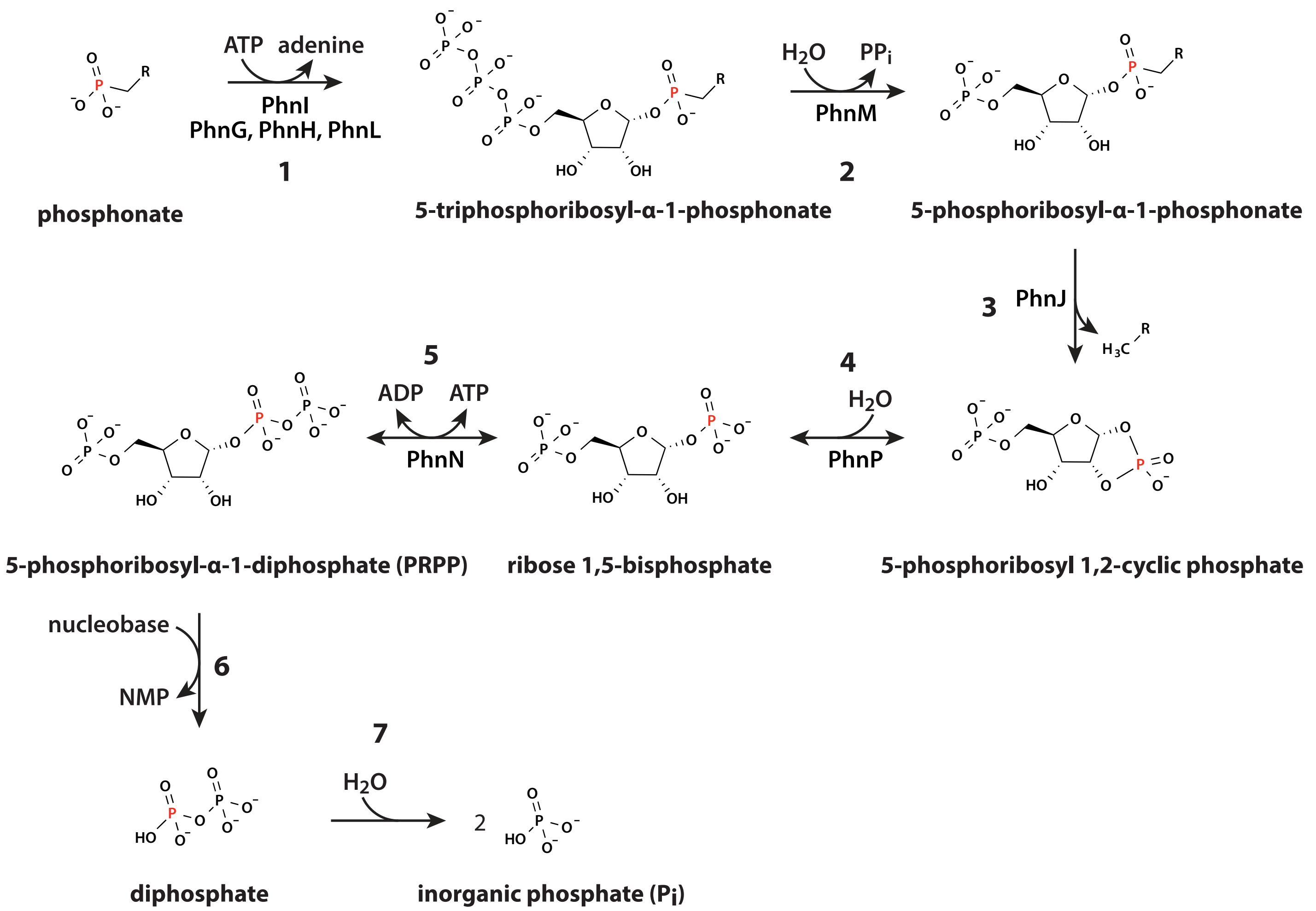

Supplementary Figure 2

a

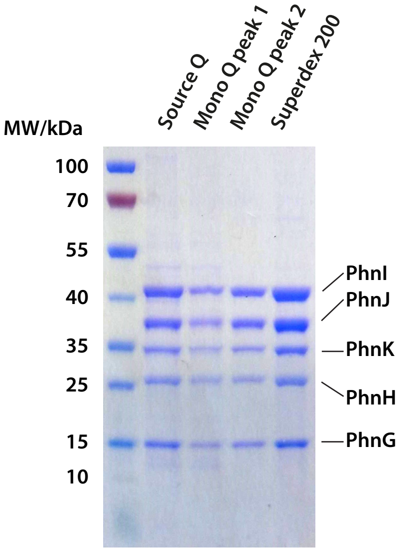

b

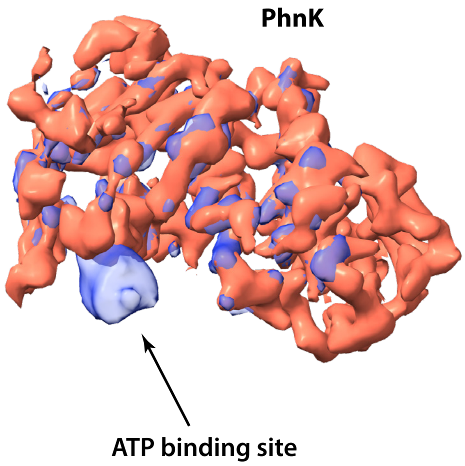

c

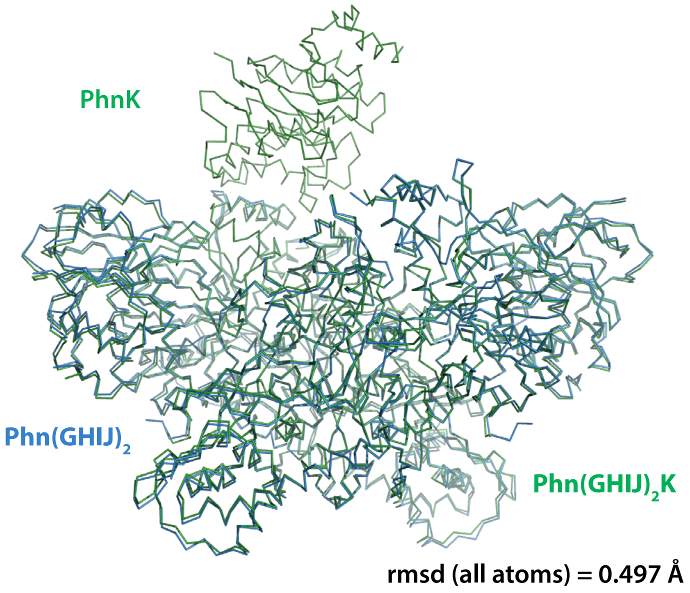

Supplementary Figure 3

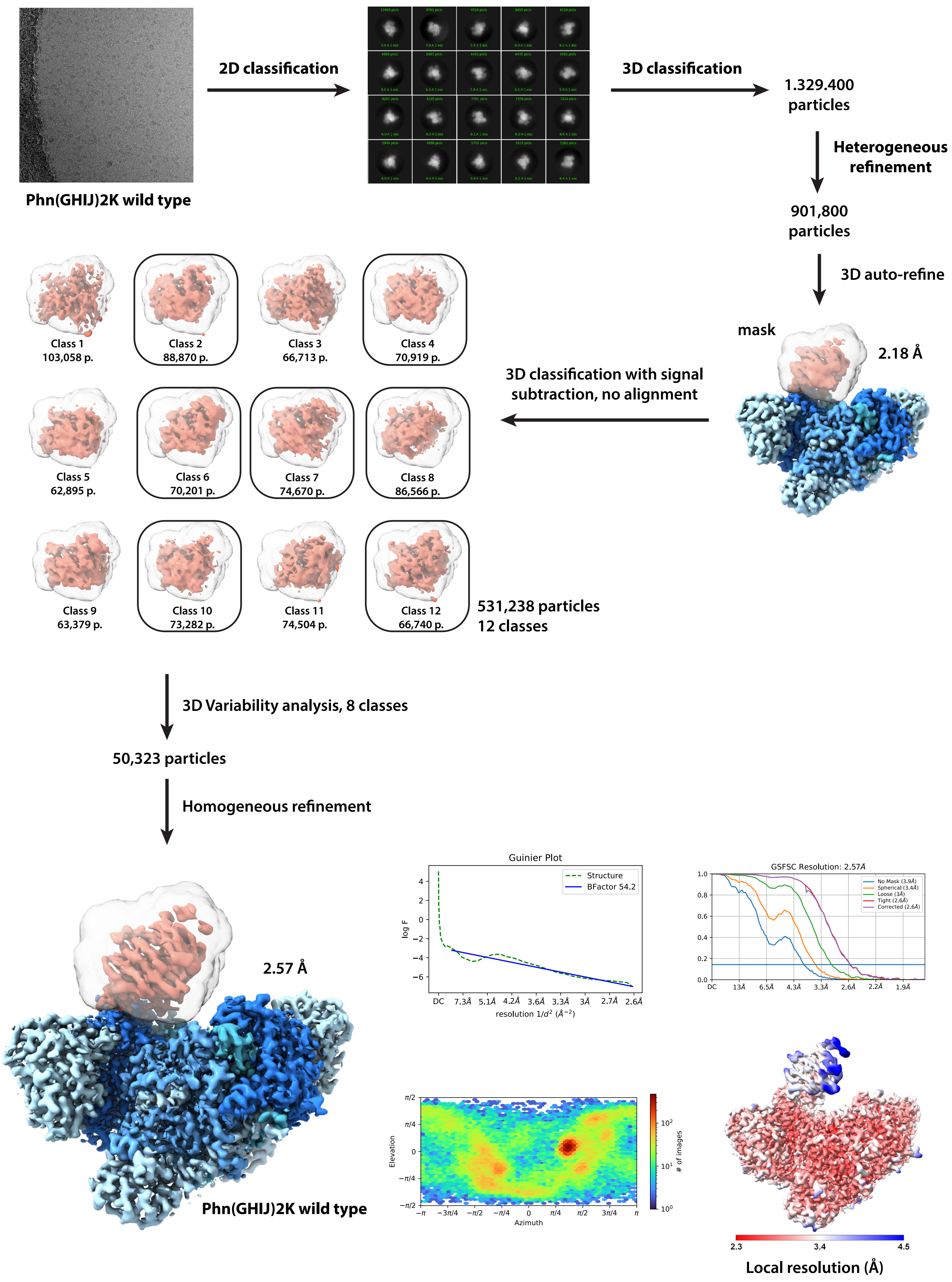

Supplementary Figure 4

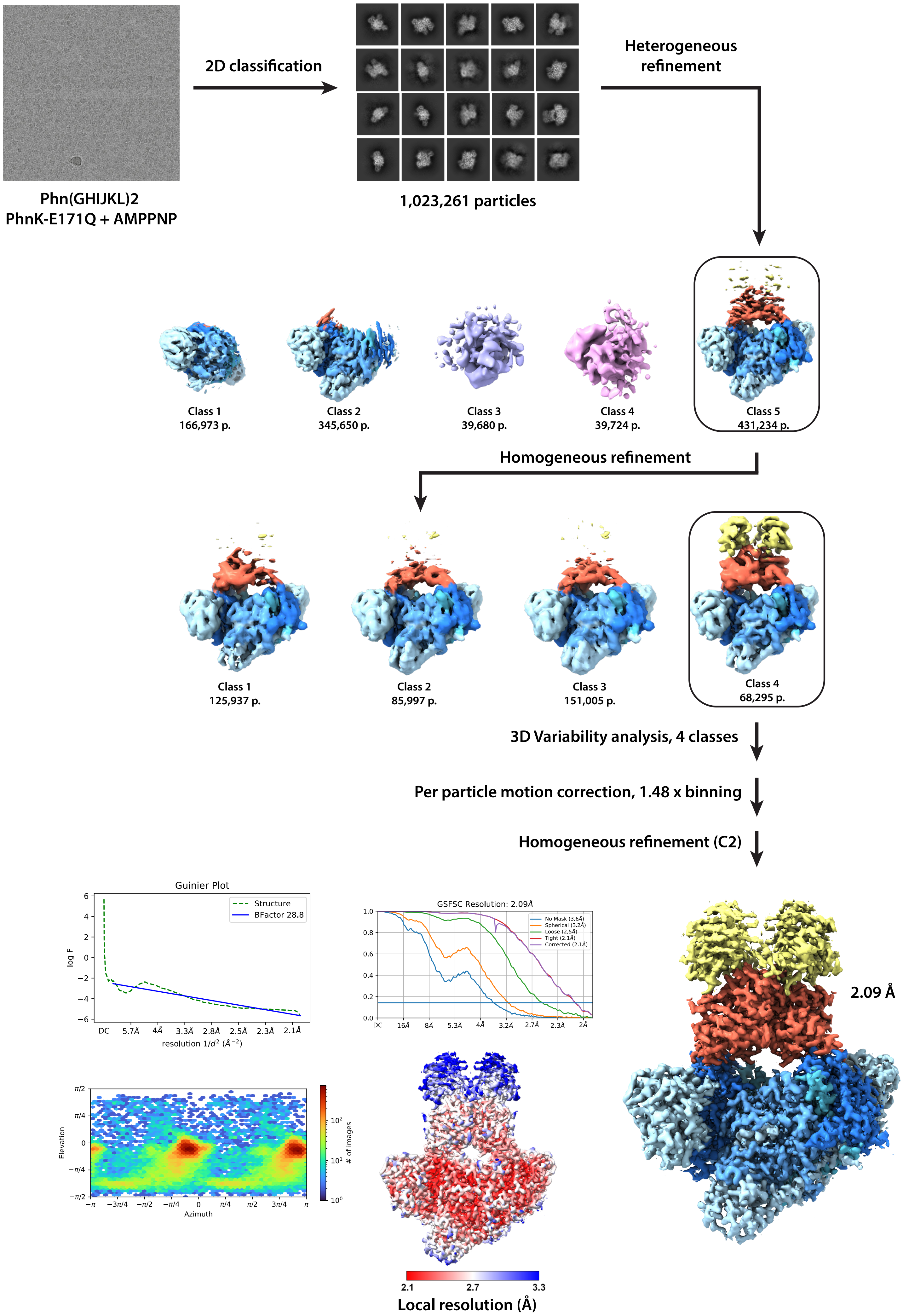

Supplementary Figure 5

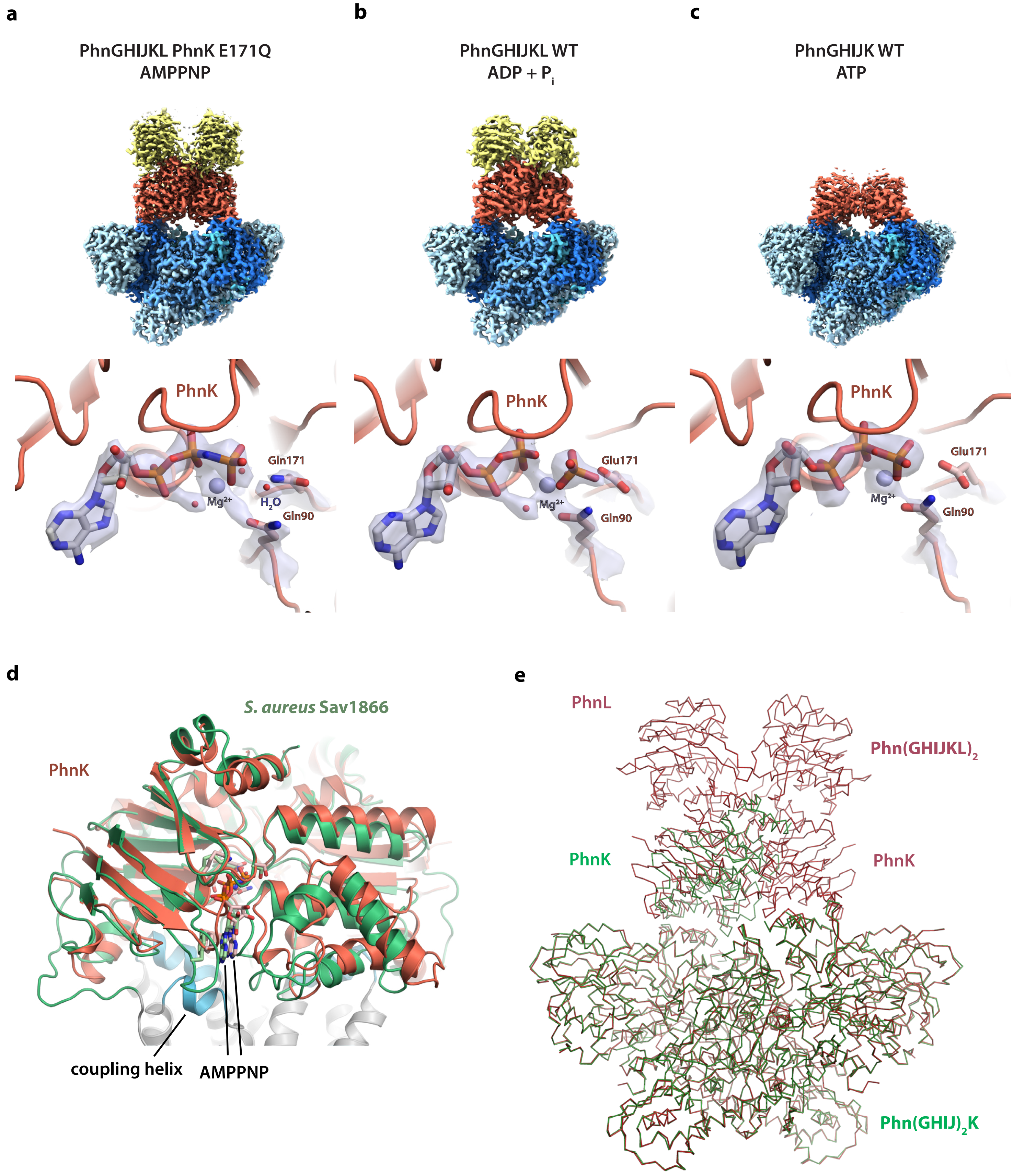

rmsd (all atoms) = 0.307 Å

Supplementary Figure 6

a

emPAI	Cov	↑*Family*	M	DB	Accession	Score	Mass	Matches	Match(sig)	Sequences	Seq(sig)	Description
16.41	0.57	1	1	Trembl	**A0A023Z6E0_ECOLX**	13332	32067	375	375	14	14	Alpha-D-ribose 1-methylphosphonate 5-phosphate C-P lyase OS=Escherichia coli O145:H28 str. RM12581 OX=1248823 GN=**phnJ** PE=3 SV=1
13.98	0.57	1	2	Trembl	**A0A6G6LIX9_ECOLX**	13200	32067	367	367	13	13	Alpha-D-ribose 1-methylphosphonate 5-phosphate C-P lyase OS=Escherichia coli OX=562 GN=**phnJ** PE=3 SV=1
6.83	0.37	1	3	Trembl	**A0A2T3V5M7_ECOLX**	11531	47038	348	348	15	15	ATP-binding cassette domain-containing protein OS=Escherichia coli OX=562 GN=C9E25_06215 (**phnK**) PE=4 SV=1
3.50	0.40	1	4	Trembl	**A0A376IAT9_ECOLX**	9646	32095	273	273	8	8	Alpha-D-ribose 1-methylphosphonate 5-phosphate C-P lyase OS=Escherichia coli OX=562 GN=**phnJ** PE=3 SV=1
13.60	0.34	1	5	Trembl	**A0A6N0FP14_ECOLX**	7645	37827	268	268	13	13	Alpha-D-ribose 1-methylphosphonate 5-triphosphate synthase subunit PhnI OS=Escherichia coli OX=562 GN=**phnI** PE=4 SV=1
6.96	0.42	1	6	Trembl	**A0A7B1JLD3_ECOLX**	4130	27813	133	133	9	9	ABC transporter ATP-binding protein OS=Escherichia coli OX=562 GN=**phnK** PE=4 SV=1
3.87	0.31	1	7	Trembl	**A0A788LHF4_ECOLX**	3507	21197	116	116	6	6	Phosphonate C-P lyase system protein PhnH OS=Escherichia coli OX=562 GN=**phnH** PE=4 SV=1
2.60	0.28	1	8	Trembl	**A0A376RQC2_ECOLX**	3327	56753	113	113	11	11	Phosphonates transport ATP-binding protein PhnL OS=Escherichia coli OX=562 GN=**phnL** PE=4 SV=1
2.88	0.26	1	9	Trembl	**A0A7B7R490_ECOLX**	2963	21228	97	97	5	5	Phosphonate C-P lyase system protein PhnH OS=Escherichia coli OX=562 GN=**phnH** PE=4 SV=1
2.10	0.26	1	10	Trembl	**A0A7B6UGW1_ECOLX**	1242	21154	40	40	5	5	Phosphonate C-P lyase system protein PhnH OS=Escherichia coli OX=562 GN=**phnH** PE=4 SV=1
4.58	0.45	1	11	Trembl	**A0A5C9AAH9_ECOLX**	1181	16686	31	31	5	5	ATP-binding cassette domain-containing protein (Fragment) OS=Escherichia coli OX=562 GN=FWK02_34920 (**phnL**) PE=4 SV=1
3.15	0.29	1	12	Trembl	**A0A418GIX9_ECOLX**	1146	16803	38	38	4	4	ATP-binding cassette domain-containing protein OS=Escherichia coli OX=562 GN=D3C88_16455 (**phnK**) PE=4 SV=1
63.30	0.23	1	13	Trembl	**A0A7A1M1F4_ECOLX**	648	8967	42	42	3	3	Carbon-phosphorus lyase complex subunit **PhnI** (Fragment) OS=Escherichia coli OX=562 GN=HJ332_004423 PE=4 SV=1
55.86	0.61	2	1	Trembl	**A0A376TPT2_ECOLX**	4221	15267	160	160	9	9	Phosphonate metabolism protein OS=Escherichia coli OX=562 GN=**phnG** PE=4 SV=1
12.18	0.43	2	2	Trembl	**A0A449CRE9_ECOLX**	3552	16642	133	133	7	7	Carbon-phosphorus lyase complex subunit OS=Escherichia coli OX=562 GN=**phnG** PE=4 SV=1
0.66	0.13	3	1	Trembl	**A0A023Z718_ECOLX**	273	57464	7	7	6	6	60 kDa chaperonin OS=Escherichia coli O145:H28 str. RM12581 OX=1248823 GN=groL PE=3 SV=1
0.33	0.06	4	1	Trembl	**A0A023Z3R1_ECOLX**	64	16733	1	1	1	1	Biotin carboxyl carrier protein of acetyl-CoA carboxylase OS=Escherichia coli O145:H28 str. RM12581 OX=1248823 GN=accB PE=4 SV=1
0.17	0.05	5	1	Trembl	**A0A023YYV7_ECOLX**	58	30545	1	1	1	1	Protein MtfA OS=Escherichia coli O145:H28 str. RM12581 OX=1248823 GN=mtfA PE=3 SV=1
0.32	0.07	6	1	Trembl	**A0A023Z5Q5_ECOLX**	53	17464	1	1	1	1	Regulator of ribonuclease activity A OS=Escherichia coli O145:H28 str. RM12581 OX=1248823 GN=menG PE=3 SV=1
0.63	0.09	7	1	Trembl	**A0A0E0U6W5_ECOLX**	49	9656	1	1	1	1	Uncharacterized protein OS=Escherichia coli UMNK88 OX=696406 GN=UMNK88_5065 PE=4 SV=1
0.07	0.02	8	1	Trembl	**A0A023YRN2_ECOLX**	47	69130	1	1	1	1	Chaperone protein DnaK OS=Escherichia coli O145:H28 str. RM12581 OX=1248823 GN=dnaK PE=2 SV=1

b

PhnK dimer (top side)

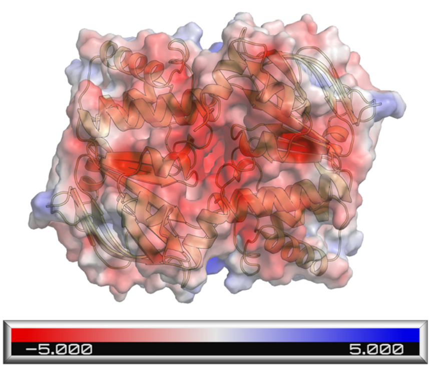

PhnL dimer (underside)

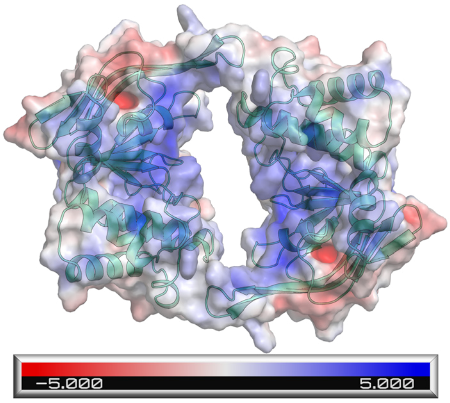

c

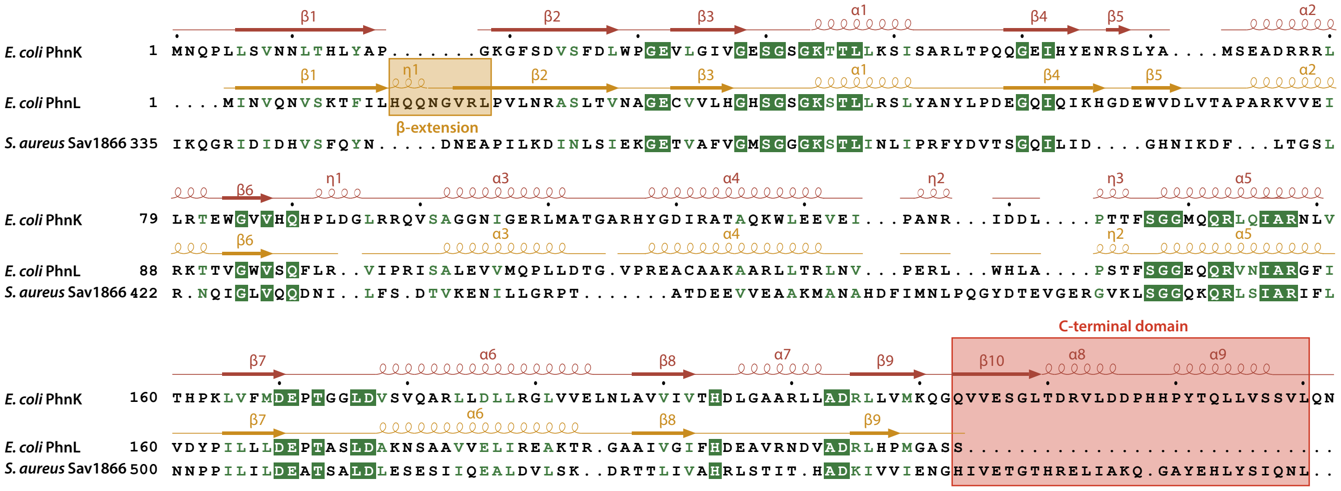

Supplementary Figure 7

a

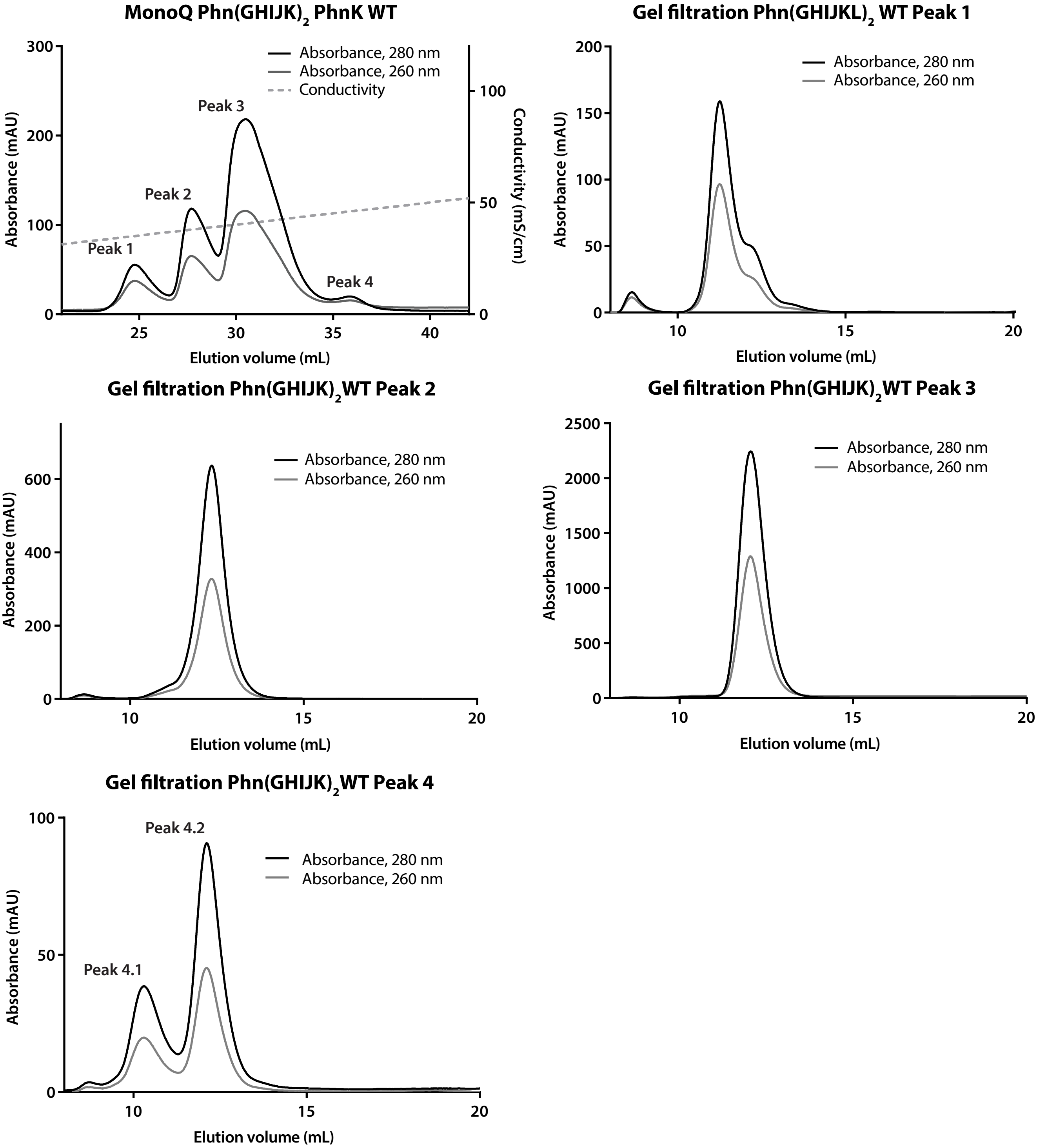

b

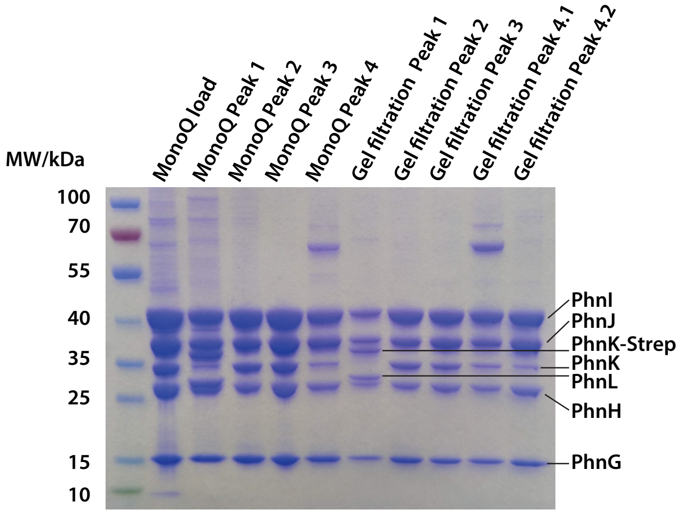

c

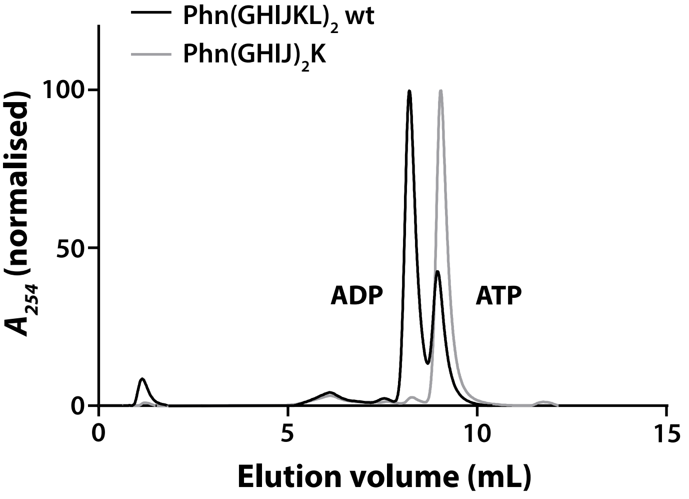

Supplementary Figure 8

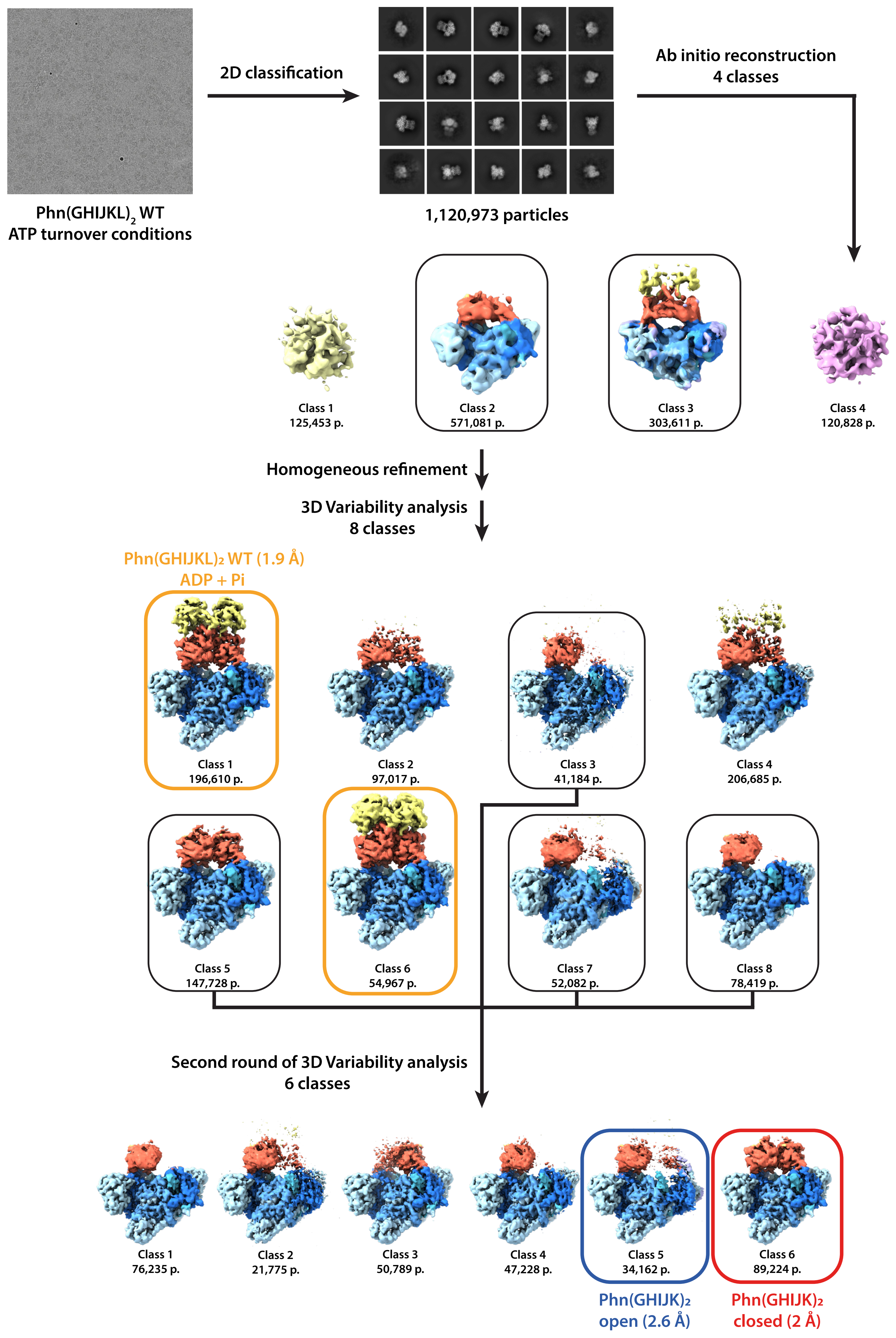

Supplementary Figure 9

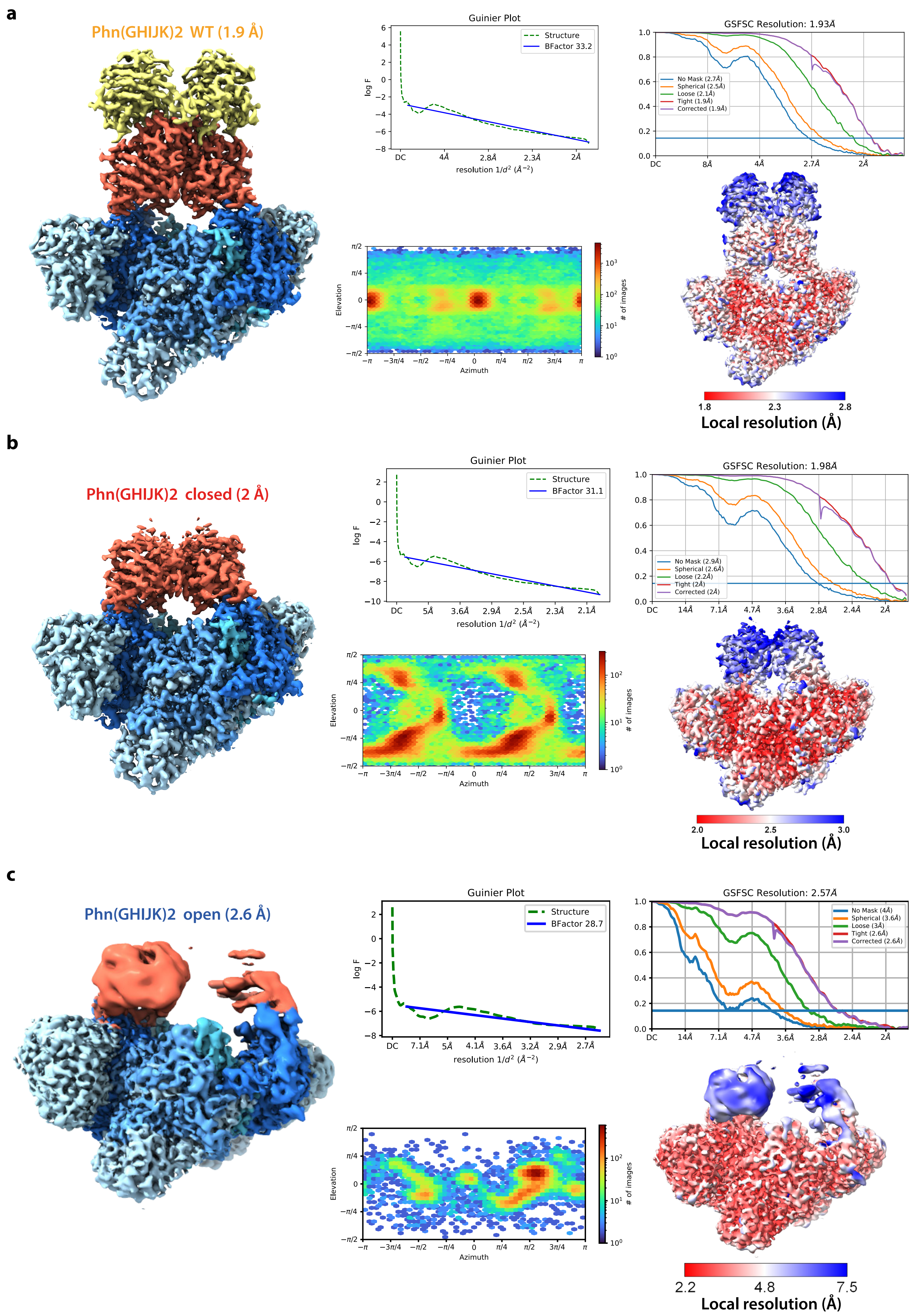

Supplementary Figure 10

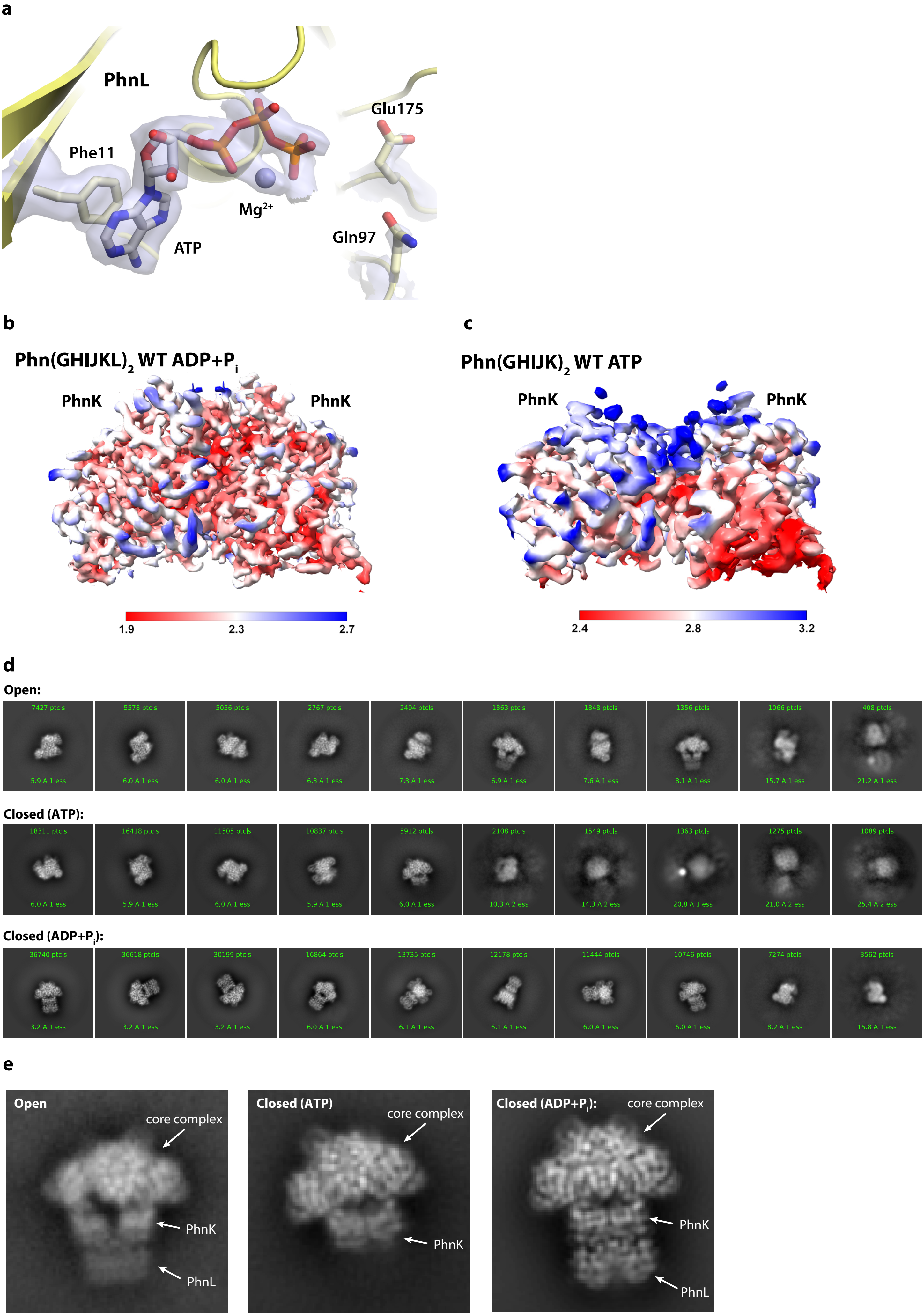

Supplementary Figure 11

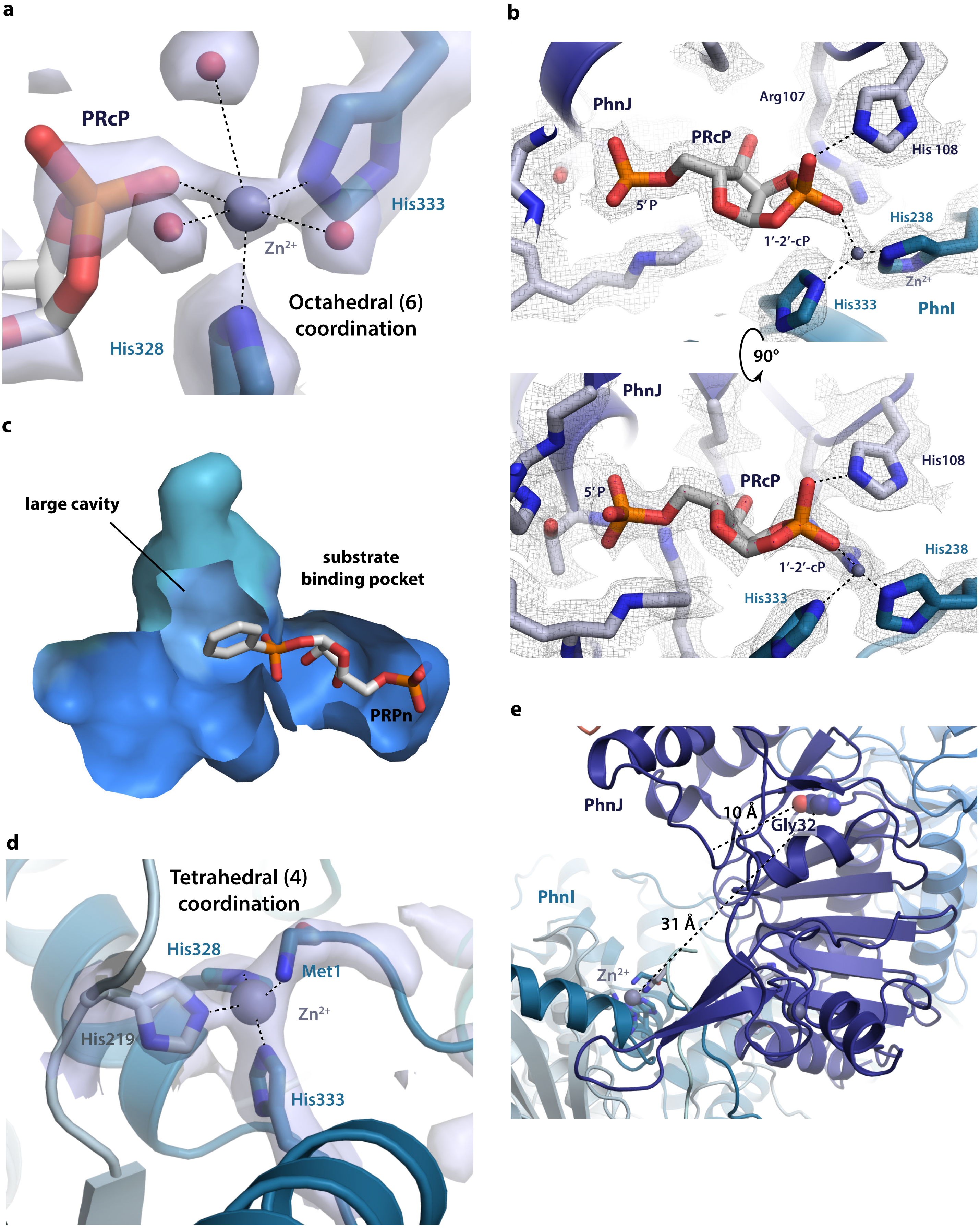

Supplement: Supplementary file 1 — Supplementary Information [file 41467_2023_36604_MOESM1_ESM.pdf]
