# Supplementary material for: Structural remodelling of the carbon–phosphorus lyase machinery by a dual ABC ATPase
Source: Nat Commun. 2023 Feb 22;14:1001. doi: 10.1038/s41467-023-36604-y (PMC9947105; doi:10.1038/s41467-023-36604-y)
Supplement: Supplementary file 3 — Reporting Summary [file 41467_2023_36604_MOESM3_ESM.pdf]

nature portfolio

Corresponding author(s): BRODERSEN, D. E.

Last updated by author(s): Jan 16, 2023

Reporting Summary

Nature Portfolio wishes to improve the reproducibility of the work that we publish. This form provides structure for consistency and transparency in reporting. For further information on Nature Portfolio policies, see our Editorial Policies and the Editorial Policy Checklist.

Statistics

For all statistical analyses, confirm that the following items are present in the figure legend, table legend, main text, or Methods section.

n/a	Confirmed	
☐	☒	The exact sample size (n) for each experimental group/condition, given as a discrete number and unit of measurement
☐	☒	A statement on whether measurements were taken from distinct samples or whether the same sample was measured repeatedly
☒	☐	The statistical test(s) used AND whether they are one- or two-sided<br>*Only common tests should be described solely by name; describe more complex techniques in the Methods section.*
☒	☐	A description of all covariates tested
☒	☐	A description of any assumptions or corrections, such as tests of normality and adjustment for multiple comparisons
☐	☒	A full description of the statistical parameters including central tendency (e.g. means) or other basic estimates (e.g. regression coefficient) AND variation (e.g. standard deviation) or associated estimates of uncertainty (e.g. confidence intervals)
☒	☐	For null hypothesis testing, the test statistic (e.g. F, t, r) with confidence intervals, effect sizes, degrees of freedom and P value noted<br>*Give P values as exact values whenever suitable.*
☒	☐	For Bayesian analysis, information on the choice of priors and Markov chain Monte Carlo settings
☒	☐	For hierarchical and complex designs, identification of the appropriate level for tests and full reporting of outcomes
☒	☐	Estimates of effect sizes (e.g. Cohen's d, Pearson's r), indicating how they were calculated

Our web collection on statistics for biologists contains articles on many of the points above.

Software and code

Policy information about availability of computer code

Data collection	SerialEM 3.7 and EPU (ThermoFisher) for data collection<br>relion_it.py and cryoSPARC Live (as implemented in RELION-3/3.1 and cryoSPARC 3.0/3.1) for monitoring data collection
Data analysis	RELION-3, RELION-3.1, and cryoSPARC 3.0 and 3.1 for single-particle EM analysis<br>MotionCor2 for motion correction<br>Phyre 2.0 for initial structure prediction<br>Coot 0.9 and ISOLDE 1.3 for molecular model building.<br>NAMDINATOR 1.0 for structure geometry improvement.<br>Phenix-1.19.2 for real space refinement.<br>UCSF ChimeraX-1.3 and PyMOL 1.0 for data analysis and presentation.

For manuscripts utilizing custom algorithms or software that are central to the research but not yet described in published literature, software must be made available to editors and reviewers. We strongly encourage code deposition in a community repository (e.g. GitHub). See the Nature Portfolio guidelines for submitting code & software for further information.

Data

Policy information about availability of data

All manuscripts must include a data availability statement. This statement should provide the following information, where applicable:

- Accession codes, unique identifiers, or web links for publicly available datasets
- A description of any restrictions on data availability
- For clinical datasets or third party data, please ensure that the statement adheres to our policy

The structural data generated in this study have been deposited in the Protein Data Bank and EM Data Bank under accession codes 7Z19 [http://doi.org/ 10.2210/pdb7z19/pdb] and EMDB-14445 [https://www.ebi.ac.uk/emdb/EMD-14445] (Phn(GHIJ)2K), 7Z16 [http://doi.org/10.2210/pdb7z16/pdb] and EMDB-14442 [https://www.ebi.ac.uk/emdb/EMD-14442] (Phn(GHIJKL)2 PhnK-E171Q:AMPPNP), 7Z15 [http://doi.org/10.2210/pdb7z15/pdb] and EMDB-14441 [https://www.ebi.ac.uk/emdb/EMD-14441] (Phn(GHIJKL)2 WT:ADP + Pi), 7Z18 [http://doi.org/10.2210/pdb7z18/pdb] and EMDB-14444 [https://www.ebi.ac.uk/emdb/EMD-14444] (Phn(GHIJK)2 WT:ATP closed), and 7Z17 [http://doi.org/10.2210/pdb7z17/pdb] and EMDB-14443 [https://www.ebi.ac.uk/emdb/EMD-14443] (Phn(GHIJK)2 WT:ATP open).
4XB6 [http://doi.org/10.2210/pdb4XB6/pdb] (Phn(GHIJ)2)
2ONJ [http://doi.org/10.2210/pdb2ONJ/pdb] (S. aureus Sav1866 multidrug transporter ABC domain)

Human research participants

Policy information about studies involving human research participants and Sex and Gender in Research.

Reporting on sex and gender	Not relevant as there are no human research participants.
Population characteristics	Not relevant as there are no human research participants.
Recruitment	Not relevant as there are no human research participants.
Ethics oversight	Not relevant as there are no human research participants.

Note that full information on the approval of the study protocol must also be provided in the manuscript.

Field-specific reporting

Please select the one below that is the best fit for your research. If you are not sure, read the appropriate sections before making your selection.

☒ Life sciences ☐ Behavioural & social sciences ☐ Ecological, evolutionary & environmental sciences

For a reference copy of the document with all sections, see nature.com/documents/nr-reporting-summary-flat.pdf

Life sciences study design

All studies must disclose on these points even when the disclosure is negative.

Sample size	Set to allow identification of possible outliers.
Data exclusions	One data point was excluded in Figure 3c (negative activity due to high background).
Replication	The cryo-EM data are inherently replicated as they represent the averages of 100,000s of particles.<br>In vivo and biochemical experiments were replicated at least twice as stated in the figure legends.
Randomization	Not relevant as there are no experimental groups
Blinding	Not relevant as there are no experimental groups.

Reporting for specific materials, systems and methods

We require information from authors about some types of materials, experimental systems and methods used in many studies. Here, indicate whether each material, system or method listed is relevant to your study. If you are not sure if a list item applies to your research, read the appropriate section before selecting a response.

Materials & experimental systems

n/a	Involved in the study
☒	☐ Antibodies
☒	☐ Eukaryotic cell lines
☒	☐ Palaeontology and archaeology
☒	☐ Animals and other organisms
☒	☐ Clinical data
☒	☐ Dual use research of concern

Methods

n/a	Involved in the study
☒	☐ ChIP-seq
☒	☐ Flow cytometry
☒	☐ MRI-based neuroimaging
